# Surgical Treatment of Palatal Defects

**Published:** 1901-05-15

**Authors:** Truman W. Brophy


### SURGICAL TREATMENT OF PALATAL DEFECTS.

BY DR. TRUMAN W. BROPHY.

Extracts from an arti le read at the International Dental Congress, and published in the April *Dental Cosmos*.

It is to the credit of the dental profession that the first operation for the closure of cleft palate was proposed by La Monier, a French dentist, in 1764. And five years later Roux, another Frenchman, published methods of procedure for this operation, which he successfully carried out. In 1820 an American dentist, Warren, of Boston, without knowledge of Roux's work, published a similar method, though somewhat modified, which was generally adopted by American surgeons for the next twenty-five years. In 1844 Sir William Fergusson, of London, advocated the division of the palatal muscles to prevent the cutting out of the sutures from the great tension. In 1860 Agnew, of Philadelphia, suggested that it was the tensor palati muscle that caused the sutures to cut through, and he divided these muscles at their constricted parts, as they pass over the hamular process, thus relaxing the tension.

These operations cause a thickening and stiffening of the soft palate because of cicatricial tissue, and some of the muscles will never reunite, causing deformity of function in all the tissues implicated. Besides, the operation is difficult and often unsuccessful.

To overcome these defects the author devised an operation for adults which consists in separating the soft tissues from the bone of the vault of the mouth and dropping them down until their edges can be brought together. They are then trimmed and brought together by means of wire sutures fastened to plates of lead adapted to the surface of the tissues and at considerable distance from the cleft. By this means the edges are approximated and then drawn into close co-aptation by silk ligatures at sufficient intervals to insure continuous contact.

The advantages claimed are, that no muscles are divided, and therefore no interference with palatal function; the sutures will not cut out because the parts are held rigidly by the lead plates or splints and wire ligatures, which are secure in the entire length of the palate, and also prevent distortion by palatal muscle activity. To lengthen the palate, the palato-pharyngeal muscles may be partially split and the sections from each side brought together in the median line and united with sutures to make any desirable extension of the soft palate.

Notwithstanding the fact that eminent surgeons advise postponing operations until after the fifth or sixth year, Dr. Brophy is strongly favorable to very early operations. After making five hundred and seventy operations, of which two hundred and eleven were upon children younger than six months, the remainder varying in age from six months to fifty-two years of age, he is confident that, "*the best time for an operation is within three months after birth.*" He has had no fatalities in any operation made on children under six months of age, and the only fatalities in all these operations happened to children who were three years of age, and one of these can safely be attributed to careless after-treatment.

The author's method of operating upon children younger than three months is to draw the maxillary bones together by means of strong wire ligatures passed through the maxillary bones from the buccal side, where they are secured to lead plates, which act as non-irritant stay attachments. After drawing the bones together so as to close the cleft, the edges of the soft tissues are pared and co-apted with suitable ligatures and the parts maintained until complete union has taken place.

The advantages claimed for early operations are:

*First.*—There is less shock experienced by the patient as its nervous and sense organisms are not completely developed.

*Second.*—The bones are elastic and can be bent without fracture.

*Third.*—The palatal muscles and other tissues are brought into functional activity, and develop normally instead of atrophying.

*Fourth.*—By uniting the palatal processes of the maxillary bones the teeth develop in proper place for normal occlusion with the teeth of the lower jaw.

*Fifth.*—In early operations there is less deformity from the adhesions and contractions of scar tissue.

*Sixth.*—Early operations preclude the habit of acquiring faulty speech.

